# Pulmonary Blastomycosis: A Case from Africa

**DOI:** 10.1100/tsw.2008.141

**Published:** 2008-11-02

**Authors:** Sana Cheikh Rouhou, Hajer Racil, Olfa Ismail, Sonia Trabelsi, Mourad Zarrouk, Nawel Chaouch, Sawssen Hantous, Samira Khaled, Faouzi El Mezni, Abdellatif Chabbou

**Affiliations:** ^1^ Pulmonology Department, A.Mami Hospital, Ariana, Tunisia; ^2^ Pathology Department, A.Mami Hospital, Ariana, Tunisia; ^3^ Mycology and Parasitology Department, Charles Nicolle Hospital, Tunis, Tunisia; ^4^ Radiology Department, A.Mami Hospital, Ariana, Tunisia

**Keywords:** pulmonary mycosis, blastomycosis, lung

## Abstract

Pulmonary blastomycosis is an uncommon pathologic condition that is quite rare in Africa compared to endemic regions of Canada and the upper Midwest of the U.S. We describe a 45-year-old patient who complained of productive cough, hemoptysis, and dorsal rachiodynia. Chest imaging revealed a necrotic tissue-density pulmonary mass involving both the upper and lower right lobes. Chest MRI showed signal abnormality of the third thoracic vertebral body and the greater trochanter, consistent with metastatic lesions. Clinical and radiological findings were strongly suggestive of lung cancer. Diagnosis of pulmonary blastomycosis was made by visualization of yeast in bronchial biopsies and further confirmed by culture of bronchoalveolar lavage specimens. The patient was treated with itraconazole and his clinical condition improved markedly. Pulmonary blastomycosis is unusual in Africa and that fact caused a considerable delay in diagnosis. We suggest that this disease may be more common in Africa than has been previously suspected.

## INTRODUCTION

Blastomycosis is an endemic systemic mycosis in different parts of the world, particularly in south central and midwestern U.S.[1], but occurs sporadically in Africa. The disease is caused by a dimorphic fungus called *Blastomyces dermatitidis (B. dermatitidis)* and can involve many different organ systems[2], although the lung is almost always the organ initially infected[3]. Infection is acquired via inhalation into the lungs of airborne spores from disturbed contaminated soil where they transform into yeasts and elicit a characteristic pyogranulomatous response[4]. We report a new case of pulmonary and vertebral blastomycosis from Tunisia.

## CASE REPORT

A 45-year-old male of Tunisian origin, with a 50 pack per year smoking history, presented to our hospital for productive cough and hemoptysis. He denied fever and night sweats. On presentation, he complained of dorsal rachiodynia and lower right limb pain. He had been self-employed as a gardener at a farm for 20 years. During the execution of his work, which included spreading fertilizer and disturbing soil, he came into contact with several animal species, such as dogs, ducks, and parrots. The patient had never been to a foreign country.

The patient was febrile at 38°C with a blood pressure of 136/68 mmHg. His physical examination revealed clubbing, tachycardia at 90 beats per minute, and a diminished vesicular murmur in the right upper pulmonary field. There was no cervical or supraclavicular lymphadenopathy, no cyanosis, and no skin lesions. Routine blood tests were normal and serum was negative for HIV. Chest radiography showed a heterogeneous retractile opacity of the apical right field. Sputum microscopy and culture for mycobacteria was negative. Tuberculin skin test was nonreactive. Fiberoptic bronchoscopy showed a bulging mucosa at the entry of the upper right lobe bronchus. Histology of the bronchial cytology and biopsies were inflammatory. CT scanning of the chest revealed a necrotic tissue-density pulmonary mass involving both the upper and lower right lobes. Radiography of the dorsal vertebral column and the pelvis were normal. Bone scintigraphy disclosed an increased fixation of both the third and fourth thoracic vertebral bodies (T3 and T4), and the femur. Chest MRI showed signal abnormality of the T3 vertebral body and the greater trochanter, consistent with metastatic lesions. Flexible bronchoscopy re-examination with bronchial biopsies and bronchoalveolar lavage was performed, and revealed a histological study presence of a granulomatous material with giant cells containing spherical inclusions. These inclusions were best seen after hematoxylin-eosin (Fig. 1) and PAS (Fig. 2) stain, revealing a typical round *B. dermatitidis* yeast cell with a thick double refractile cell wall and surrounded by granulomatous tissue. Gomori methenamine silver stain showed thick-walled and broad-based budding cells with large daughter cells corresponding to reproducing *B. dermatitidis* cells (Fig. 3). Based on this characteristic histopathological appearance, blastomycosis was diagnosed. Culture of the bronchial wash on Sabouraud's dextrose agar at 25°C further confirmed the presence of *B. dermatitidis* as a fluffy white mold (Fig. 4). Serological testing for blastomycosis was negative.

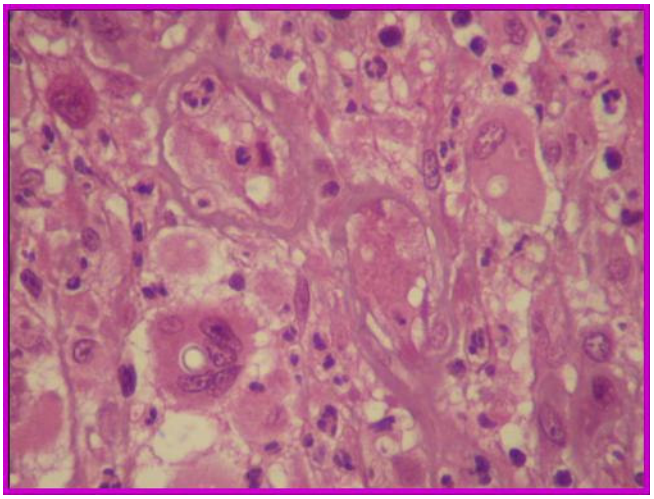

**FIGURE 1.** *B. dermatitidis* in bronchial biopsy. Photomicrograph (original magnification ×100, hematoxylin-eosin stain) shows a typical round *B. dermatitidis* yeast cell with a thick double refractile cell wall and surrounded by granulomatous tissue.

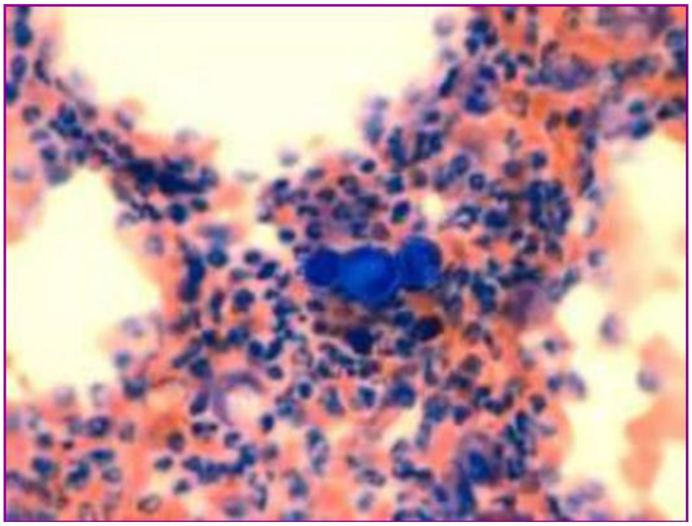

**FIGURE 2.** *B. dermatitidis* in bronchial biopsy. (Photomicrograph, original magnification ×100, PAS stain).

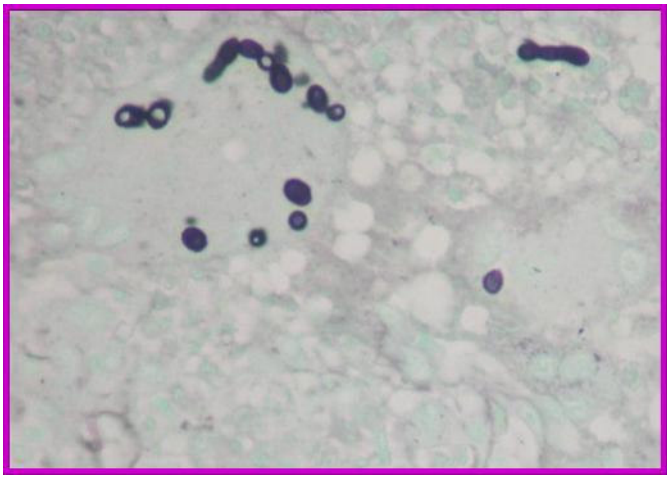

**FIGURE 3.** Bronchial biopsy. Photomicrograph (original magnification ×100; Gomori methenamine silver stain) shows reproducing *B. dermatitidis* cells, with broad-based budding and large daughter cells.

Therapy with oral itraconazole 400 mg daily commenced. The treatment was continued for 11 months. Assessment revealed improvement of the patient's clinical condition with gain of weight. Cough and hemoptysis were absent after 2 months, and thoracic pain after 3 months. Chest imaging revealed absence of radiological abnormalities.

Five months after treatment cessation, the patient complained of a reappearing right thoracic pain located in the inferior extremity of the right scapula, and general signs of fever and loss of weight. Physical examination showed painful palpation of the T3 and T4 vertebral bodies. Chest imaging showed a refractile alveolar opacity of the right upper pulmonary field, osteolytic lesion of both the second and fourth vertebral bodies, and the great trochanter. The patient was again treated with itarconazole 400 mg daily. The patient is now following the treatment course and assessment after 4 weeks revealed improvement of thoracic pain and general signs.

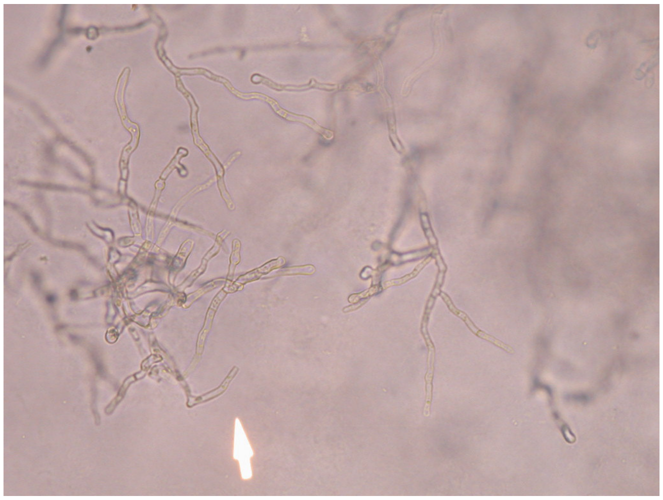

**FIGURE 4.** Culture on Sabouraud's dextrose agar at 25°C. *B. dermatitidis* growing in a mycelial form as a fluffy white mold. The branching hyphae are 2 to 3 µm in diameter and have right-angled conidiophores that have a single terminal conidia.

## DISCUSSION

Blastomycosis is a chronic infection characterized by granulomatous and suppurative lesions. The disease is caused by *B. dermatitidis*, a dimorphic fungus. It exists in mycelial form in nature and as yeast at body temperature in tissue. The conidia produced from the hyphae of the mycelial form are infectious for humans.

Blastomycosis has been sporadically found in Africa and the Centers for Disease Control and Prevention estimate the incidence in endemic areas at one or two cases per 100,000 population[3]. Few cases of pulmonary blastomycosis have been reported from different African countries[5,6,7,8,9,10]. The exact reservoir of *B. dermatitidis* is poorly defined, but is thought to be the soil. The ecology of the fungus remains obscure. Human blastomycosis is acquired through inhalation of aerosolized conidia. Rain, dew, or mist may play a critical role in the liberation of conidia, which are then dispersed. Warm soil temperature may also play a role[11,12]. Interhuman transmission has not been described[2]. The incubation period is approximately 30–45 days[6]. The pathogenesis is similar to that of histoplasmosis. Inhalation of spores results in a primary pulmonary infection. The spores are inoculated into the alveolar cells and germination to yeast forms occurs. Although pulmonary macrophages eliminate the majority of spores before infection occurs, those that survive produce pneumonitis. From the lung, there may be hematogenous spread and, less commonly, lymphatic spread. The immune response to infection consists of neutrophilic macrophage migration into the infected tissue, inducing a granulomatous and suppurative response with associated necrosis and fibrosis characteristic of blastomycosis[13].

Pulmonary blastomycosis is considered by some authors as a "great masquerader"[14]. It can present in several ways as either acute or chronic disease, and clinical manifestations can range from asymptomatic infection to a fulminant clinical course. The ability to mimic other diseases is a hallmark of blastomycosis, including bacterial pneumonia, malignancy, and tuberculosis, and often leads to erroneous or delayed treatment[3,15,16]. Clinical symptoms typically include cough, fever, weight loss, and chest pain[1,4]. Hemoptysis may occur[2,15]. Radiologic manifestations of pulmonary infection are very diverse and mainly include airspace consolidation, mass-like lesions, interstitial and miliary infiltration, and cavitary lesions[1,3,15].

It is generally accepted that the primary site of infection is the lung, with subsequent dissemination to other sites. Extrapulmonary disease may involve one or several body sites, the most common being the skin, followed by bone, the male genitourinary system, and, less commonly, the central nervous system (CNS)[1,3]. Osseous blastomycosis, as described in our case, occurs in up to 25% of extrathoracic cases[2]. Bone involvement affects most frequently the vertebrae, pelvis, sacrum, skull, ribs, and long bones[2,13], and is the result of hematogenous dissemination or direct invasion from adjacent structures[17]. The most commonly affected parts of the spine include the lumbar and thoracic region[18]. Bone destruction may lead to wedge-shaped compression fractures or gross deformities[19]. Areas of osseous involvement usually appear as lucent (radiography) or low attenuation (CT) lesions with indistinct margins, but have no radiologically specific features to help distinguish them from other forms of osteomyelitis[15]. Blastomycosis of the vertebral spine may be confused with vertebral tuberculosis[20].

Occurrence of hemoptysis on presentation, and presence of both a necrotic tissue-density pulmonary mass and metastatic-like bone lesions on skeletal imaging, strongly suggested the hypothesis of a pulmonary carcinoma with bone metastasis in our case.

To confirm blastomycosis, *B. dermatitidis* must be isolated in culture of sputum or bronchial aspirate specimen, or observed on histological examination of tissue obtained by biopsy (bronchus, lung, skin, and prostate). Diagnosis is made from observing the characteristic thick-walled, broad-based budding yeast after special stains[21]. The organisms can be recovered on Sabouraud's dextrose agar at room temperature as a fluffy white mold.

Serological testing for blastomycosis infection, including complement fixation antibodies and immunoprecipitines, is not reliable due to poor sensitivity and false-positive results from cross-reactivity with other fungi[22]. The serologic testing using an enzyme immunoassay for detection of antibody to the A antigen of *B. dermatitidis* can help to diagnosis and yield useful results to distinguish acute from chronic disease[23,24].

Spontaneous resolution of chronic blastomycosis does not appear to occur and untreated disease can be associated with mortality rates approaching 60%[2]. Treatment of blastomycosis has expanded with the availability of the azole antifungals, namely ketoconazole, itraconazole, and fluconazole. To date, however, no randomized blinded studies comparing different regimens have been performed. The published treatment recommendations for blastomycosis are based on relatively small, open-label controlled trials, case series, and anecdotal experience[25]. Itraconazole, at doses ranging from 200 to 400 mg daily, is considered to be the drug of choice for patients with non–life-threatening, non-CNS blastomycosis because it is associated with a better tolerance and effectiveness, with less toxicity than ketoconazole[26]. For patients with severe, life-threatening, or CNS disease, amphotericin B deoxycholate remains the drug of choice

In conclusion, our report draws attention to growing clinical experience with blastomycosis in Africa. Health care workers should be alert to the presence of this fungal infection and should appropriately consider blastomycosis in their practice.

## ACKNOWLEDGMENTS

We would like to acknowledge and thank the TheScientificWorldJOURNAL Editor.
